# Application of botulinum toxin in maxillofacial field: Part II. Wrinkle, intraoral ulcer, and cranio-maxillofacial pain

**DOI:** 10.1186/s40902-019-0224-2

**Published:** 2019-10-16

**Authors:** Kyung-Hwan Kwon, Kyung Su Shin, Sung Hee Yeon, Dae Gun Kwon

**Affiliations:** Department of Oral and Maxillofacial Surgery, College of Dentistry, Wonkwang University, Iksan, South Korea

**Keywords:** Botulinum toxin, Clinical application, Maxillofacial field, Wrinkle, Oral ulcer, Maxillofacial pain

## Abstract

Botulinum toxin (BTX) is used in various ways such as temporarily resolving muscular problems in musculoskeletal temporomandibular disorders, inducing a decrease in bruxism through a change in muscular patterns in a patient’s bruxism, and solving problems in patients with tension headache. And also, BTX is widely used in cosmetic applications for the treatment of facial wrinkles after local injection, but conditions such as temporomandibular joint disorders, headache, and neuropathic facial pain could be treated with this drug. In this report, we will discuss the clinical use of BTX for facial wrinkle, intraoral ulcer, and cranio-maxillofacial pain with previous studies and share our case.

## Background

In oral maxillofacial surgery, the frequency of use of botulinum toxin is increasing rapidly. Particularly, it is used not only for symptomatic treatment for a disease but also used as a method of causative treatment approach. Botulinum toxin is used in various ways such as temporarily resolving muscular problems in musculoskeletal temporomandibular disorders, inducing a decrease in bruxism through a change in muscular patterns in a patient’s bruxism, and solving problems in patients with tension headache. It is used not only for treating these diseases but also for the cosmetic purpose to smoothen wrinkles caused by the muscle of expression of the face [1].

## Clinical use for wrinkle treatment

When using botulinum toxin in various treatments, the clinician should be thorough with the concepts and knowledge of anatomical structures. It is true that the importance of knowledge on anatomical structures to reduce side effects and increase efficacy and ability to cope with each event keeps increasing. As botulinum toxin can be used for various treatments, accordingly, it is also important to acquire knowledge on anatomical structures for various purposes [1–5].

### Anatomy of facial expression muscles

**Fig. 1:**
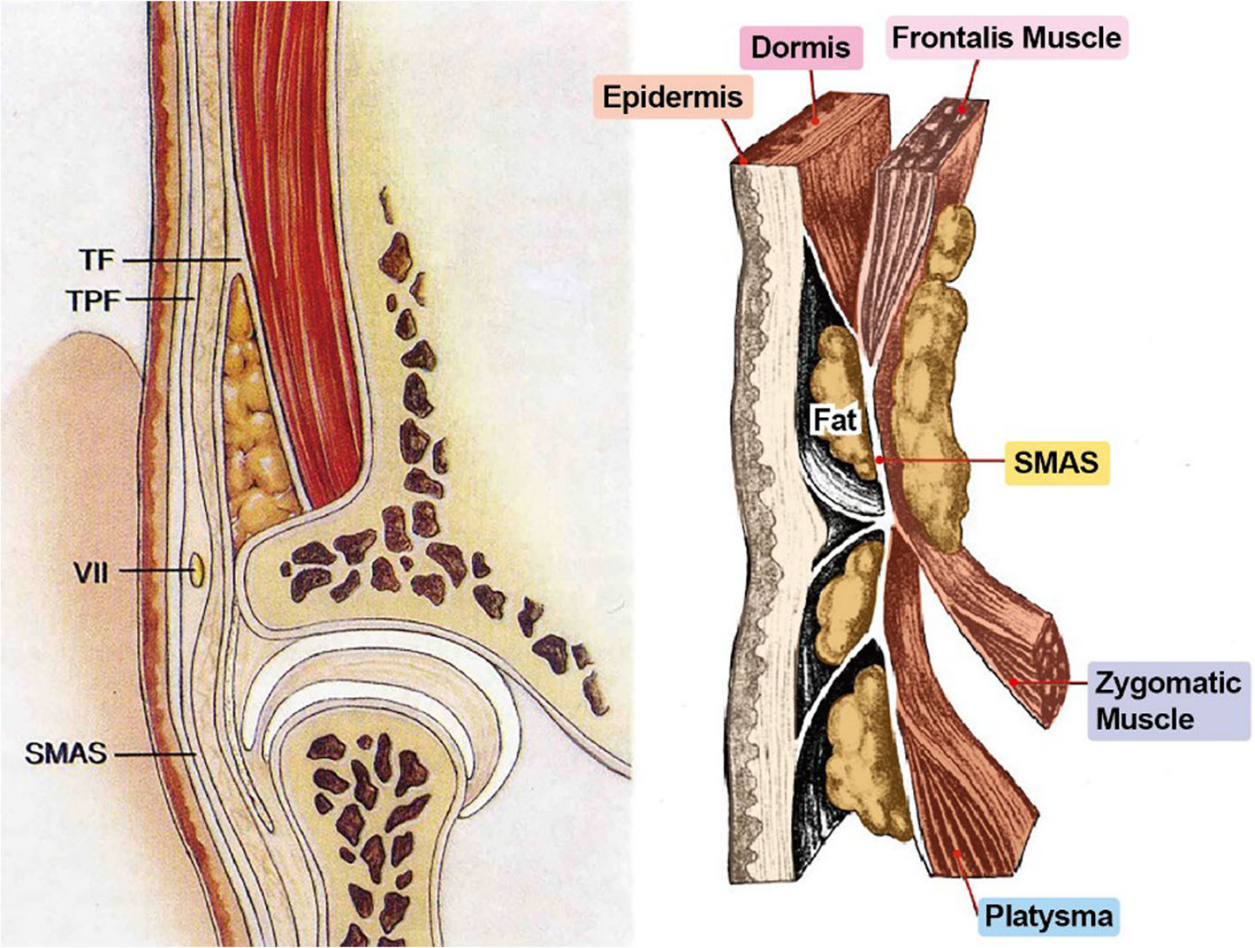
Schematic representation of superficial musculoaponeurotic system (SMAS)

The superficial musculoaponeurotic system (SMAS) layer is an important anatomical structure when acquiring anatomical knowledge for muscles of expression. When we observe the skin of the face and muscle layers beneath the skin, the head, and the neck, SMAS is connected to the dermis at one end, and the other end is connected to fascia of facial muscles. Superiorly, it is connected to temporalis muscles and frontalis muscles, anteriorly connected to orbicularis oculi, inferiorly connected to platysma, and posteriorly connected to trapezius muscles. Description of the SMAS layer is shown schematically in Fig. 1.

The SMAS layer is also used in facelift surgery, which is a procedure to lift the SMAS layer posteroinferiorly and flatten the facial wrinkles [6, 7].

**Fig. 2:**
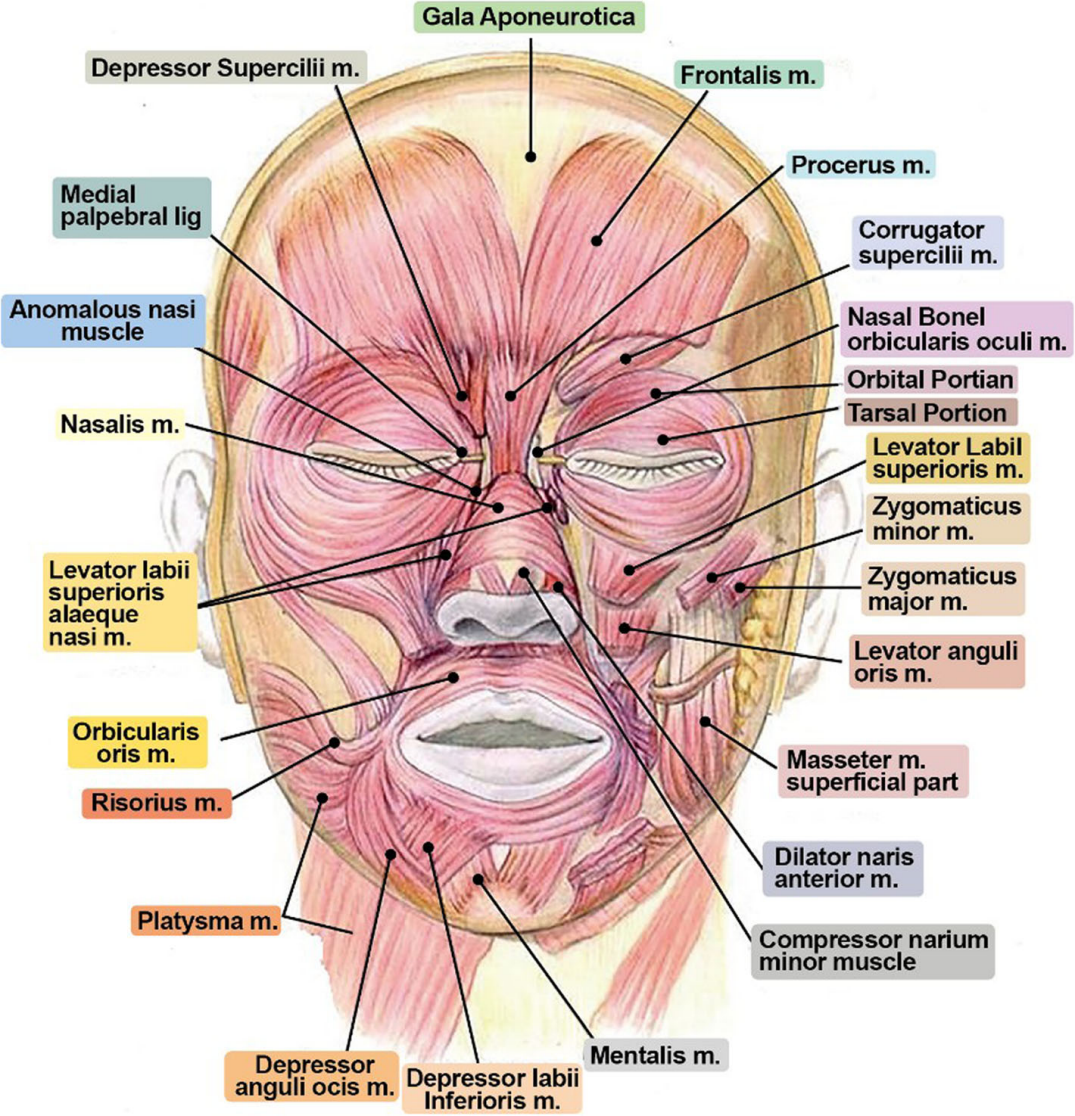
It is very important to understand the origin and insertion of expression muscles of the face in wrinkle treatment. Especially, one should carefully observe the origin and insertion of frontalis muscles, corrugator supercilii m., procerus m., orbicularis oculi m., and orbicularis oris m.

The frontalis muscle has no bony attachments since it originates from galea aponeurotica and inserts into the skin of the eyebrows (Fig. 2). Its function is to lift the skin around the nasal root, making wrinkles on the forehead. It is supplied by a temporal branch of the facial nerve and supraorbital and supratrochlear artery. It is responsible for forehead crease, and botulinum toxin injection can change the pattern of creases through flaccid paralysis of contraction of muscles. Wrinkles will re-appear after 6 months, but in a different pattern than that of the past.

Orbicularis oculi muscles is connected to the nasal part of the frontal bone and frontosphenoidal process by medial palpebral ligament and lateral palpebral ligament, respectively. It is responsible for closing of the eyes.

Corrugator supercilii is located beneath the frontalis and orbicularis oculi, and it draws the eyebrows medially downward. It is supplied by the temporal branch of the facial nerve and supreatrochlear artery. It is used as the principal target muscle during botulinum toxin injection as it is regarded as the muscle causing glabellar lines.

Risorius originates from the fascia of the masseter muscle, proceeds horizontally forward, and inserts into the skin at the corner of the mouth. Its function is to pull the corner of the mouth sideward to produce a smile. It is innervated by the buccal branch of the facial nerve and supplied by the transverse facial artery and facial artery. Injecting botulinum toxin into the orbiculis oris affects the risorius muscle leading to drooping of the lips [8].

### Treatment of glabellar lines

The proceus muscles, corrugator supercilii muscles, and depressor supercilii muscles are involved in the formation of glabellar lines. A good treatment effect is expected for wrinkles formed during the facial movement, but for deep wrinkles, using collagen matrix or injecting fillers may be recommended. In general, the effect is to last for 3–5 months. Repeated injections may not completely remove wrinkles, but once the wrinkles start to deform, you can observe the wrinkles disappearing. With every 6 months of botulinum injection, the wrinkles begin to deform, and if this persists, they will be transformed into different forms from the existing wrinkles [6, 9, 10].

**Fig. 3:**
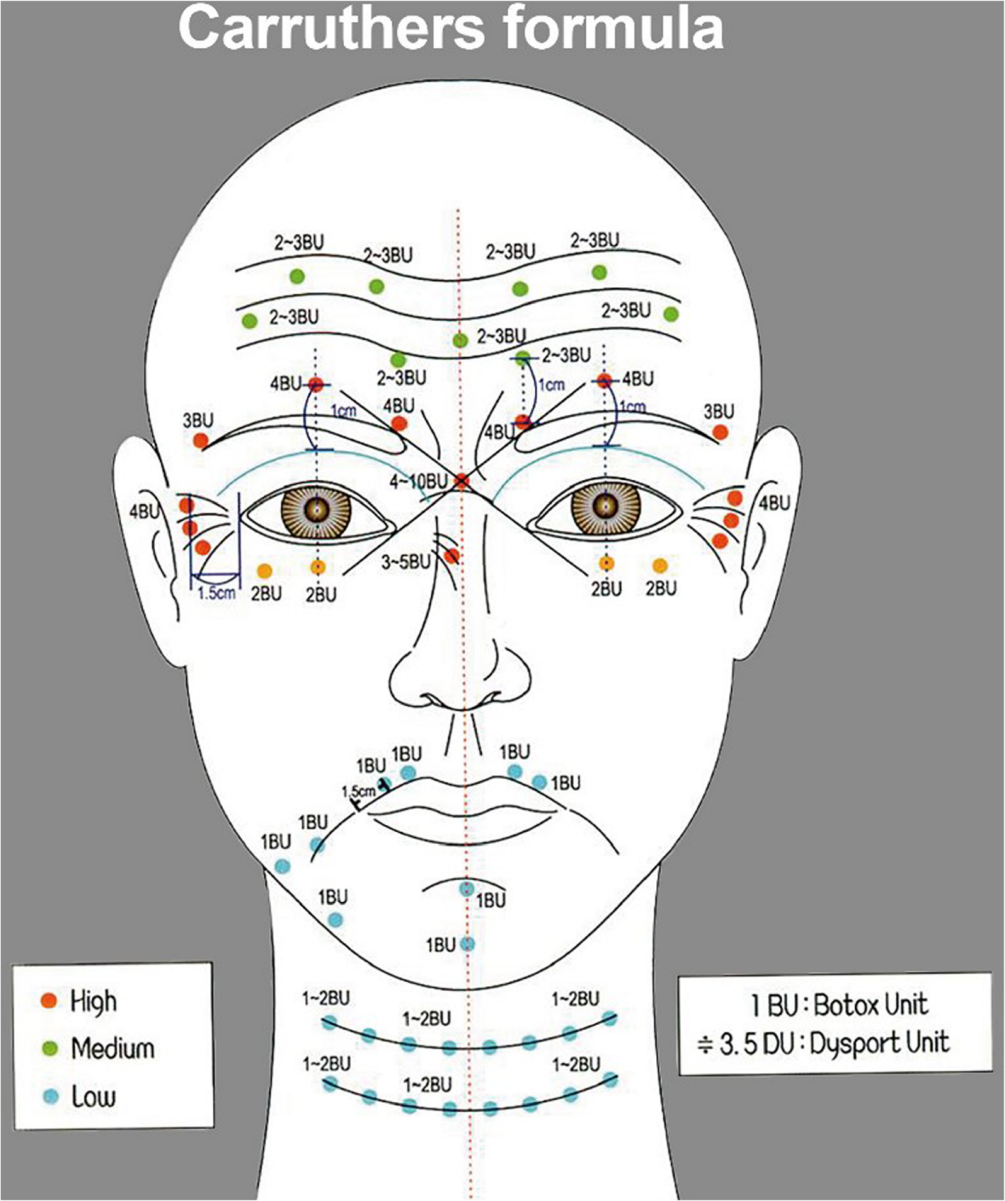
A protocol used for forehead creases and eye wrinkles. The standard dose was determined based on BTXA. (formula by Carruthers)

It is important to target the corrugator muscle while injecting botulinum toxin. In our case, we mix BTXA (Hanall pharmaceuticals) with 2.5 cc saline to make 100 units, then make 4 unit per 0.1 cc and then inject 4 units per injection point (dosage required for each part is shown in Fig. 3). We use a 60° angle and advance the needle until the bone is gently contacted. (It is different for each operator. Some operator use less than 45° and some use 90°. Choosing the degree angle is a matter of operator’s preference. Even though we randomly set the criteria for choosing the degree angle, as a matter of fact, there is a difference in the extent of diffusion depending on the degree angle.) After inserting the needle and checking the exact area, inject slightly upward direction. To reduce the effect on glabellar lines of orbicularis oculi and to cause complete paralysis of the corrugator muscle, inject 1 cm away from the orbital bony rim at the midpupillary line of the eyes. When we classify the patients based on the types of wrinkles, i.e., shallow lines, deep lines stretchable with hands, and permanent deep creases, you do not have to adjust the dose of botulinum toxin for shallow lines, but if there is a case where the patient does not want a big change in his impression, you may reduce the dose as required. Inject with 50% dose into the insufficient area after a week or two for deep lines that are stretchable with hand [11, 12].

Lastly, you may consider using fillers after injecting with 50% of the dose into the insufficient area after a week or two for the case of permanent deep creases. The patient should be aware of additional use of fillers.

Carruthers had reported that he had given additional injections to paralyze the orbicularis on the inner side of the corrugator muscle, located 1 cm above the orbital bony rim. This is why we must pre-determine the location to give an additional injection into the deficient area in a week to 2 weeks [11].

While there are not many side effects from its use, upper eyelid ptosis may be the worst side effect. It is caused by partial paralysis of the levator palpebrae superioris that resolves after 2–4 weeks. To minimize side effects, it is important not to touch or press the injection site to prevent excessive diffusion of botulinum toxin around the eyes. Also, the patient should not lie down for 2–3 h right after the injection. The operator may reduce the dose as much as possible and must keep in mind that he would not inject botulinum toxin into the area below the eyebrows exterior to the internal canthus [9, 10]

Treatment effect of glabellar lines with botulinum toxin will appear 3–4 days after the injection, and you can expect the significant effect only after 10 days of injection. Generally, after a week or two, you should decide whether the additional injection is required.

### Treatment of forehead wrinkles

Unlike Westerners, Asians, especially Koreans, have broader a frontalis muscle that extends to the edge of the forehead rather than restricted to the center of the forehead. Therefore, it is necessary to inject the whole area including the lateral portion of the forehead. If we follow Western guidelines (injecting only in the center of the forehead), it will increase the incidence of a condition called samurai eyebrows (V-shaped eyebrows). This is because the contractibility of the frontalis muscle remains which brings imbalance to the facial harmony, which eventually leads to the lifting of the eyebrows. In my early days, I also had such a case where I unintentionally caused samurai eyebrows by not injecting into the temporal muscles, but there are few such cases nowadays. The fact that large frontal bellies have no attachment point to the bone, and more of the muscles are distributed on the temporal side rather than the central area, is an important thing to remember while injecting botulinum toxin [13].

**Fig. 4:**
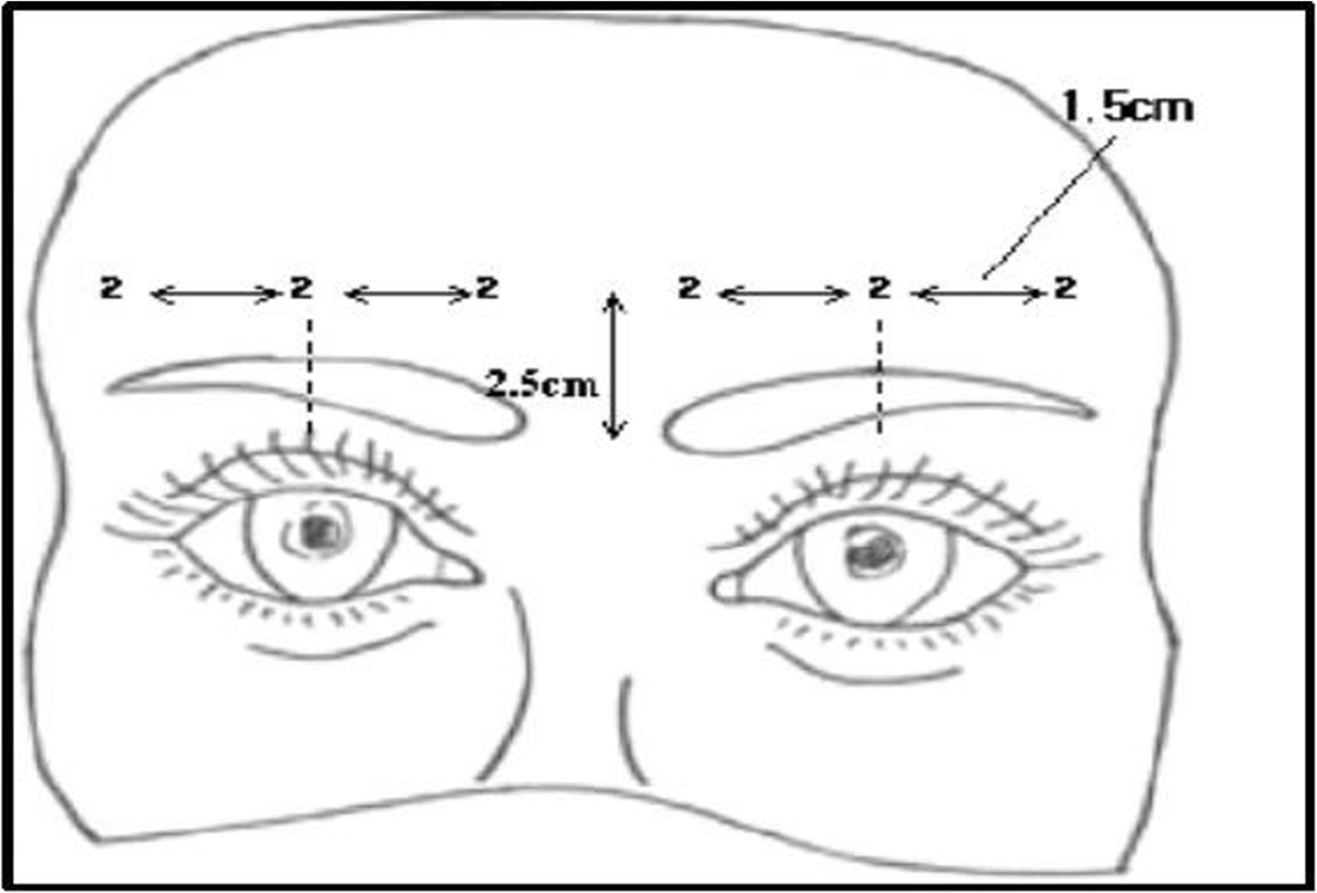
Schematic representation of injection points for forehead wrinkles. Injection point

Inject one or two lines of injecting points with a distance of 1.5 to 2.5 cm depending on the width of the forehead, the depth of the wrinkles, the number of wrinkles, and the distribution of the wrinkles. According to Khawaja, and Hassan Abbas et al., toxins spread 2.5–3 cm in all directions from the injection point in the case of the front forehead, and the effect spreads uniformly. When we inject with 2 U (0.05 cc per injection point), the range of botulinum toxin diffusion is generally about 1.5 cm in radius, 3 cm in diameter (Fig. 4).

**Fig. 5:**
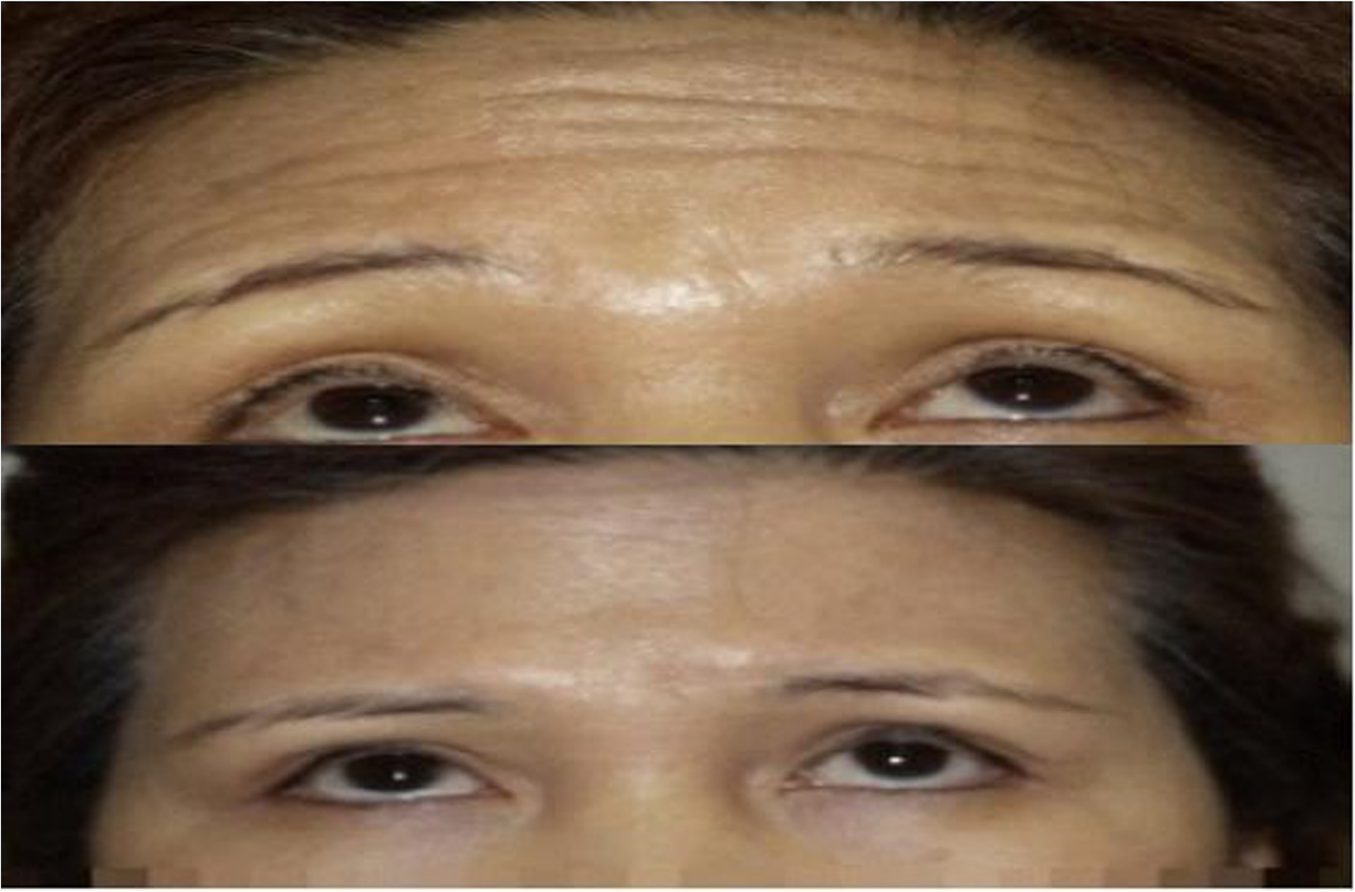
(Up) Initial diagnosis, forehead wrinkles are observed. (Down) 2 weeks after BTXA injection

In Koreans, injecting 4–10 points would be sufficient to treat both glabellar lines and forehead wrinkles. Imagine there is a vertical line drawn on the outer boundary of the eyebrows. Inject lateral to the line, both left and right sides of the face. Inject 4–6 points symmetrically with a distance of 2 cm into the horizontal line drawn 2.5 cm above the orbital rim. If there are wrinkles in the upper part of the central region, give one or two additional injections. Use 2 U per each injection (Fig. 5).

Side effects include eyebrow ptosis, upper eyelid swelling, and samurai’s eyebrows. If you comply with the standard precautions, there won, give one or two a [9, 10].

Figure 5 shows 2 weeks after the injection of BTXA into the glabellar lines. The patient tries hard to make wrinkles by raising her eyebrows. For deep wrinkles that one can spread by hand, the effect of botulinum toxin will be the most prominent.

### Treatment of eye wrinkles (angulus oculi lateralis wrinkles)

Eye wrinkles are closely related to the aging progress. After the 30s, skin elasticity decreases and smile wrinkles start to appear. As the fibroelastosis continue to progress, the wrinkles resembling crow’s feet tend to develop and this eventually is recognized as a sign of aging. The so-called crow’s feet wrinkles are considered to be caused by the intrinsic aging of the skin and the photo-aging by ultraviolet rays as well as the worsening smile lines due to the chronic and excessive contraction of the orbicularis oculi and the fibrous thickening of the orbicularis oculi muscle [14, 15].

**Fig. 6:**
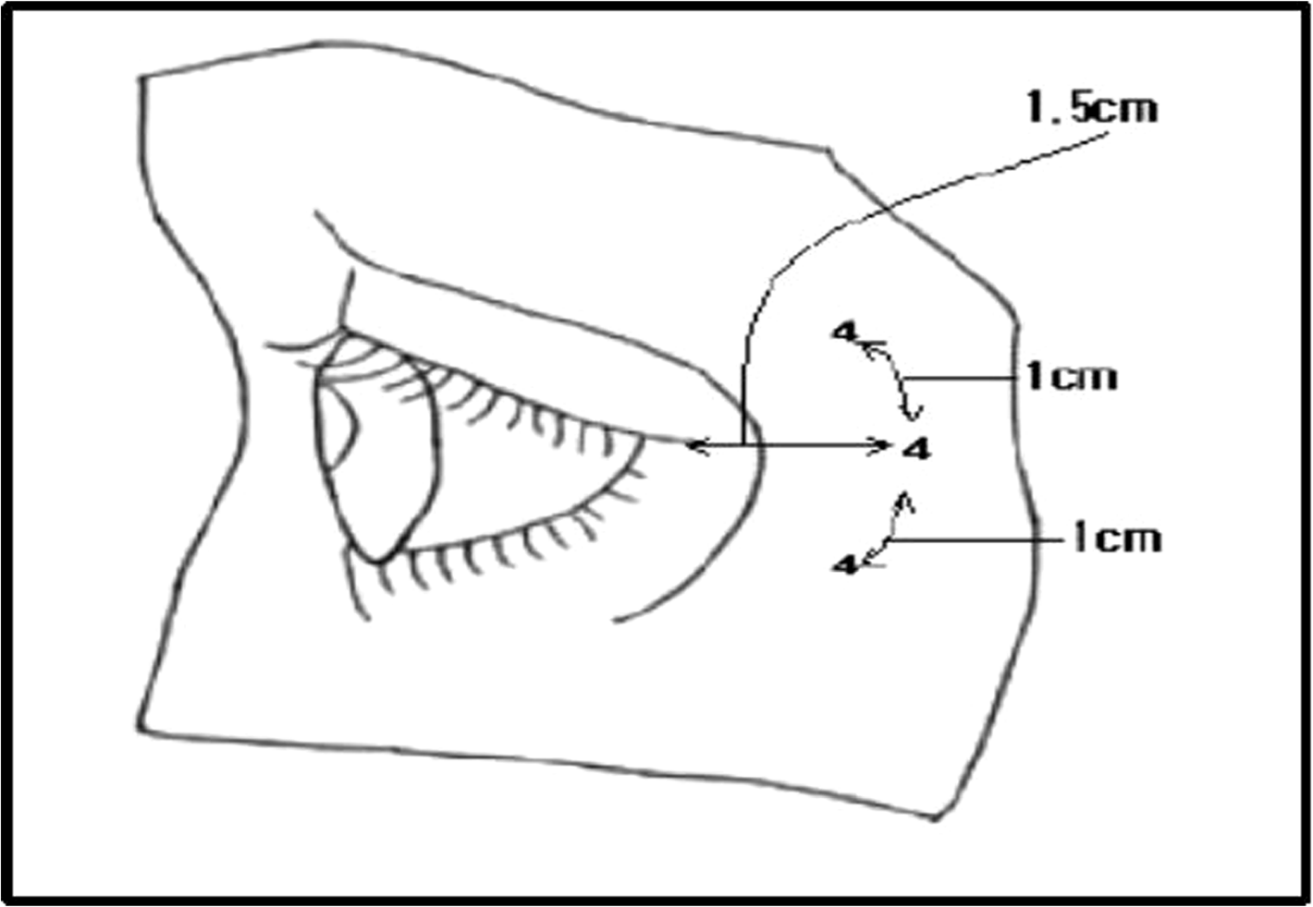
Schematic representation of injection point for eye wrinkles

It is a basic principle to inject a high-concentration–small-dose injection of botulinum toxin for the treatment of eye wrinkles. This is a precise, long-lasting method, and has fewer side effects than low-concentration–high-dose injection. Normally, 0.1 cc injection allows effective injection in 4 U increments after dilution with 2.5 cc of distilled water or saline. In general, it is essential to inject 1 cm per point 1.5 cm away from where the orbital bone is felt (Fig. 6) [11, 12].

**Fig. 7:**
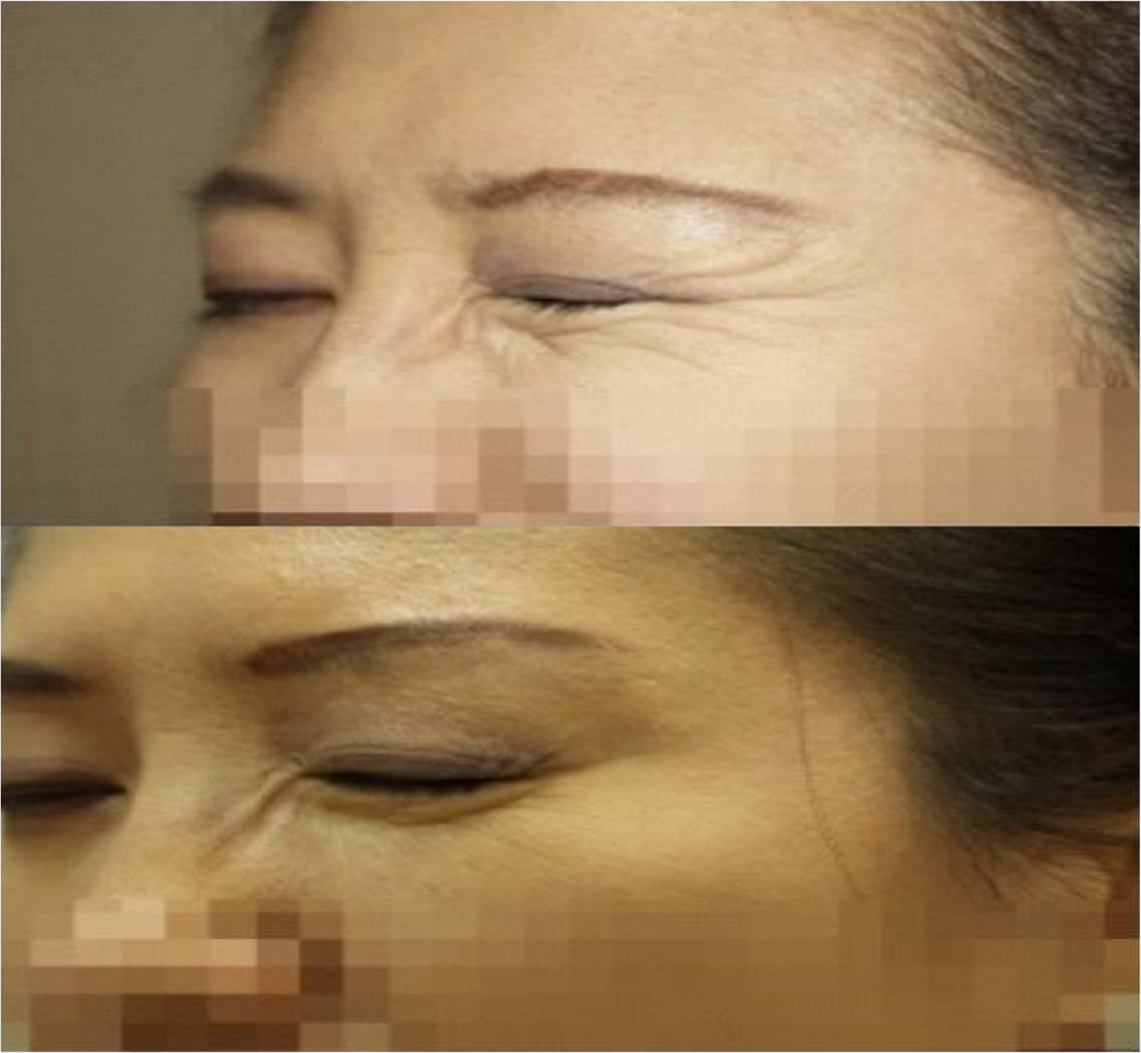
Treatment of eye wrinkles. After a week of BTXA injection, the patient is not able to make eye wrinkles even if she tries to do. (Up) Patient at initial diagnosis. (Down) 1 week after BTXA injection

It is advised to inject at a 45° angle because the skin is thin. After injection, gently rub the area toward the opposite direction of the orbit to evenly spread the toxin. As it is easy to bruise, it is also a good way to reduce the risk of bruising by shrinking blood vessels while reducing pain with local anesthetics. Make sure the patient does not lie down for 2 to 3 h after the procedure as the injection may flow to the nose or may spread to the unwanted area (Fig. 7).

## Clinical use for intraoral ulcer

### Treatment of oral ulcerative lesion using botulinum toxin

It is reported to be effective in the treatment of oral ulcers in some literature. However, more studies on clear mechanisms and efficacy seem necessary. It has been reported to be effective in anal fissure. An anal fissure is a lacerated wound of the anal canal from the anal verge to the dentate line. In the acute phase, a simple laceration becomes chronic and ulcers are formed. The cause was unknown in the past [16, 17]. Local ischemia at the anal fissure site plays an important role in the cause of disease. In such a case, when the internal anal sphincter is injected with botulinum toxin, it is known that it relaxes the muscles, thereby increasing blood circulation treating necrosis or chronic ulcers [5].

Occurrence of oral ulcer is thought to be due to various reasons, one of which is an autoimmune disease, whereas some people think that it occurs as a result of activation of the dormant virus in the peripheral nerves during the period of trauma, endocrine disorder, menstruation, and allergy, where the immune system is significantly lowered [18].

Formation of oral ulcer is based on the hypothesis that the intraoral ulcer progress from a traumatic ulcer to a chronic ulcer due to the friction between the tooth and mucosa due to the interaction between the orbiculis oris muscle and buccinator muscle. Moreover, it is also considered that viral infection in the form of opportunistic infections is additionally involved. Therefore, along with the botulinum toxin treatment, treatment of intra-oral ulcers should be considered by eliminating certain causes and improving intra-oral environments [19].

Inject only into the mucosa and not the surrounding muscles. Generally, inject 0.5 units (2.0 cc of distilled water or normal saline mixed with 100 units of BTXA, use 0.01 cc) with the idea of injecting (infiltration) into the boundary of the ulcer about 2 mm away from the center of the ulcer. Inject one point per each ulcer.

**Fig. 8:**
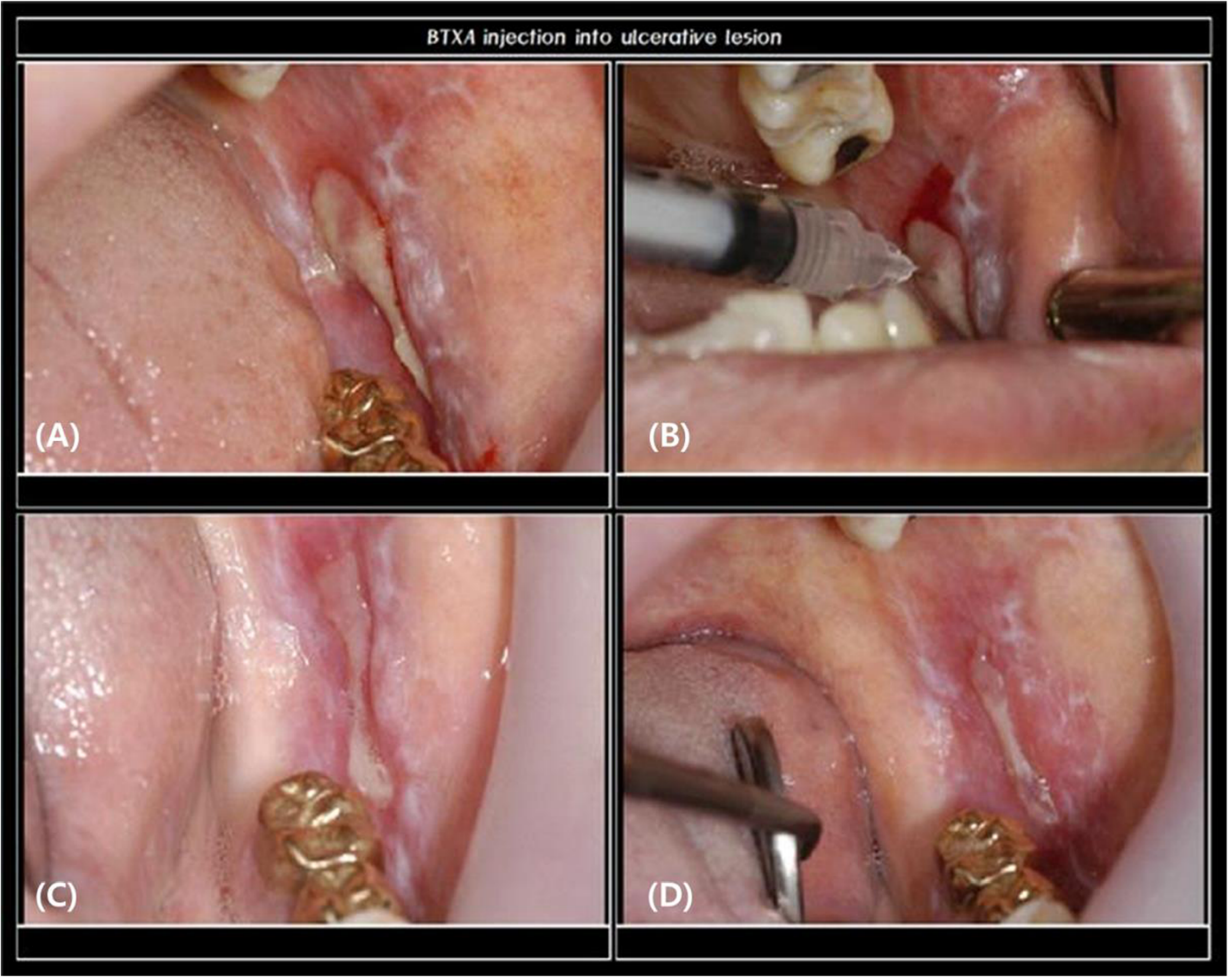
A case with oral ulcerative lesion injected with botulinum toxin. **a** Clinical intraoral photograph at first visit. **b** Botulinum toxin injected 0.5 unit. **c** 2 weeks after injection. **d** 3 weeks after injection

Figure 8 shows a case of a treatment of oral ulcer by botulinum toxin in our hospital. A patient suffered from oral ulcer persisting even though the patient visited and received treatment from the Department of Oral Medicine and the Department of Periodontology. On biopsy, the ulcerative lesion was found to be erosive lichen planus, and when we injected 0.5 unit of BTXA into the mucosa surrounding the lesion and at the center of the lesion, it relieved the pain and significantly reduced the size of the ulcer.

## Clinical use for cranio-maxillofacial pain

One of the most common symptoms of cranio-maxillo-facial pain is headache. Headache is the most common distressing symptoms regardless of the pathophysiological mechanism, morbidity rate, and causes. In literature, headache is described as one of the 10 most common symptoms in clinical medicine, and 8% of patients who visits the hospital complains of a headache [20]. It is no exaggeration to say that most of dental clinic in-patients complain of headache when we distinguish the related pain and referred pain. The principle of treating headache is the removal of the source of pain. Even if the intrinsic structural lesion does not appear in most patients who complain of headache, it is important to keep in mind of such possibilities. About one-third of brain tumor patients complain of a headache as a primary symptom.

The intensity of pain, character and area of pain, and, especially, period of headache and accompanying neurologic symptoms can give an idea of investigating the intrinsic factors. Sudden onset of severe headache is related to intracranial disorders such as subarachnoid hemorrhage or meningitis rather than chronic headache. Headache due to sleep disorder, exertional headache, and paroxysmal headache in older adults also may imply intrinsic structural lesions such as lethargy, visual or limb symptoms, convulsion, and change in the state of mentality like in the case of headache accompanied by neurologic symptoms. Although chronic headache is commonly caused by migraine, tension, or depression, it may be related to intracranial lesions, head injury, cervical spondylosis, dental or ocular disease, temporomandibular function disorder, sinusitis, hypertension, and various internal medicine disorders. Computed tomography (CT) or magnetic resonance imaging (MRI) of head, electroencephalography, and lumbar puncture test is performed based on the initial diagnosis and patient’s situation. You should diagnose and treat the primary neurologic disorder associated with headache with respect to the abovementioned diseases. However, very often, it is difficult to identify the source of pain in the case of chronic pain including referred pain and muscular disorders. We use various treatment protocols in managing chronic headache; removal of the intracranial and extracranial source is the principal method of approach. If the chronic headache persists even after the source has been removed, consider administration of drug (narcotic, non-narcotic analgesic), correction of occlusion, splint therapy, and relaxation of muscles by flaccid paralysis using botulinum toxin [21–26].

First and foremost, differential diagnosis is most important before proceeding with the treatment of chronic headache. Botulinum toxin treatment is preceded by removal of the source of pain depending on the differential diagnosis [21].

### Differential diagnosis

A lot of associations have published the system which practically classifies craniofacial pain. Particularly, classification of chronic pain (1986) of the International Association for the Study of Pain, classification of headache (1962) of the American Association for the Study of Headaches, and classification of headache (1994) of the American Association of Oral and Maxillofacial Surgery have a slight difference in the system [27, 28]. However, all of them use 8 systems of classification in common . Briefly, the pain can be classified into extracranial, intracranial, muscular, joint, neurological, causalgic, vascular, psychiatric (Table 1). We are going to make a differential diagnosis of head and neck pains based on these systems and discuss the possibilities of the use of botulinum toxin. The following are the classification and respective source of pain: extracranial structure disorder (teeth and craniofacial organs), intracranial (structures associated with the brain), muscular disorder (muscle, tendon, and connective tissue), joint disorder (bone, ligament, joint), neurologic disorder (peripheral nervous system), causalgic disorder (autonomous nervous system), vascular disorder (a vascular system), and psychiatric disorder (mental function). It is important to inspect these causes beforehand, and also diagnose diseases treatable with botulinum toxin injection.

**Table 1:** Eight practical classifications of headache occurring at cranio-maxillofacial area

Differential diagnosis groups	Cause of pain
Extracranial disorder	Dental and craniofacial organ
Intracranial disorder	Structures associated with the brain
Muscular disorder	Muscle, tendon, connective tissue
Joint disorder	Bone, ligament, joint
Neuralgic disorder	Peripheral nervous system
Causalgia disorder	Autonomous nervous system
Vascular disorder	Vascular system
Psychiatric disorder	Mental disorder

There is a limit to using botulinum toxin to treat all patients with a chronic headache. You can make a differential diagnosis based on the above classification system, and it is a basic step to start with.

The eight classifications useful in botulinum toxin treatment such as muscular disorder, joint disorder, neuralgic disorder, and causalgic disorder are reported in the literature. Borodic GE et al (2001) had reported that they observed satisfactory effect in 2 out of 3 patients after the treatment whose chief complaint was temporomandibular disorder symptoms and muscular disorder symptoms. Because most of the temporomandibular joint pain was due to masticatory muscles, Borodic GE et al. considered that the toxin was effective for such type of pain [21]. Since most of the muscular disorder, joint disorder, neuralgic disorder, causalgic disorder, and vascular disorder are accompanied by pain due to contraction of muscles, we think it is possible to use botulinum toxin in a wider range of treatment field.

Myofascial pain syndrome, myositis, fibrous myalgia, rigidity, and recurrent spasm are the typical diseases of headache caused by muscular disorders. These muscular disorders have typical trigger points in the muscle, so they can be used as a typical landmark while injecting botulinum toxin [25].

**Fig. 9:**
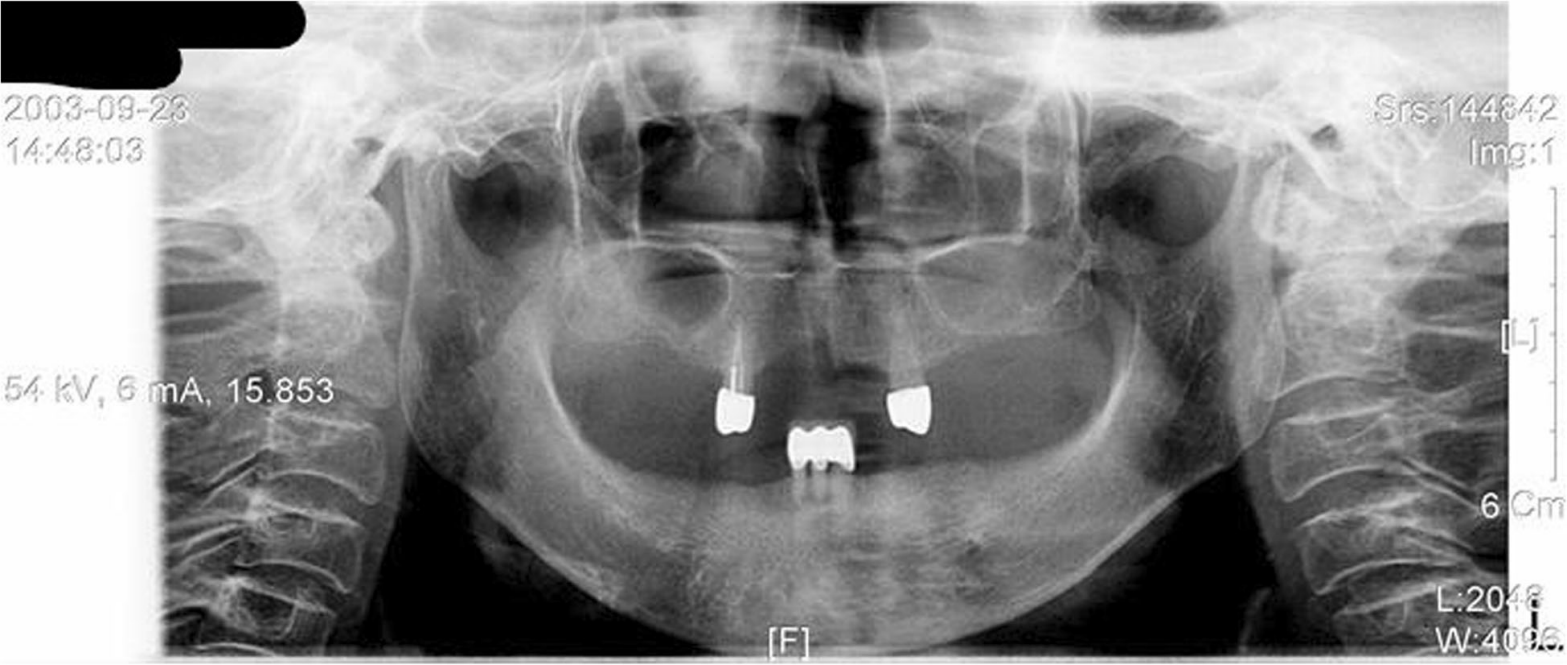
Panoramic view of a trigeminal neuralgia occurring at the right lower arch. The patient complained of neural symptoms that continued after a denture delivery, which started before the delivery of the denture. The neural symptom is paroxysmal that occurs on stimulating mental foramen

**Fig. 10:**
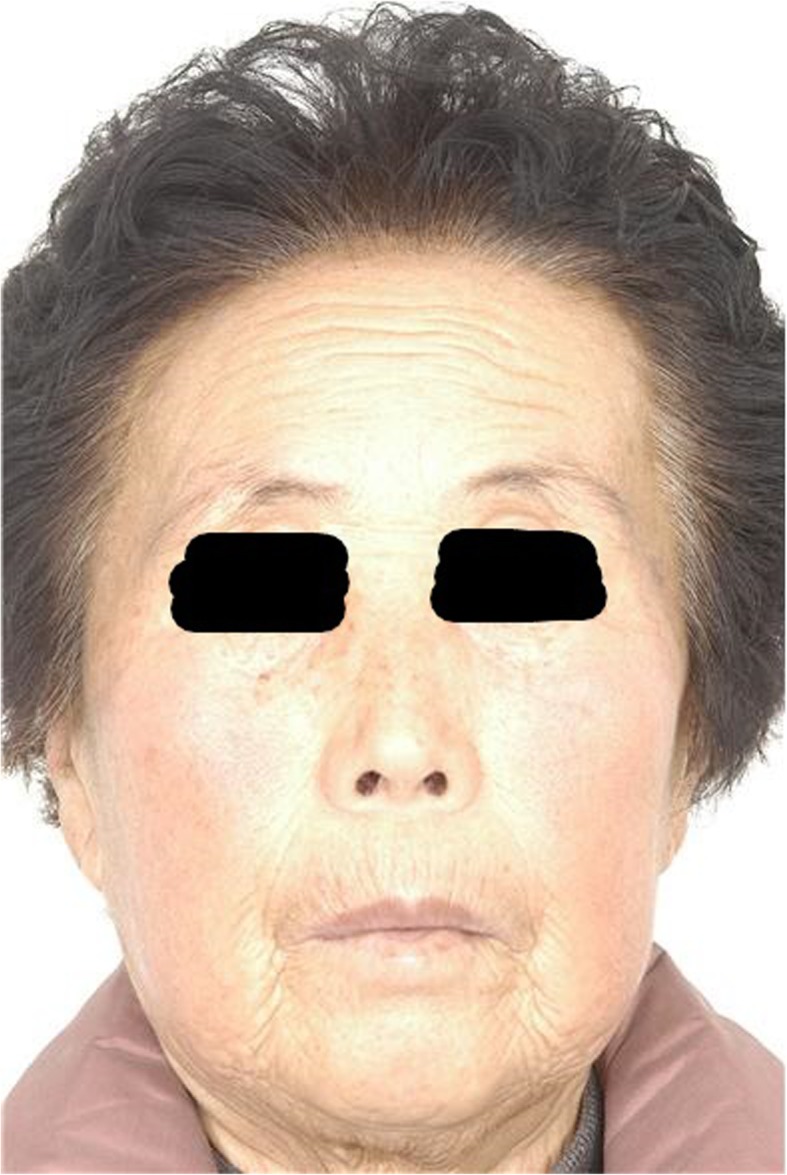
As the trigeminal neuralgia is triggered from the mental foramen area, the patient showed significant improvement when she received botulinum toxin injection around the nerves. Unfortunately, symptoms recurred after a certain period of time

**Fig. 11:**
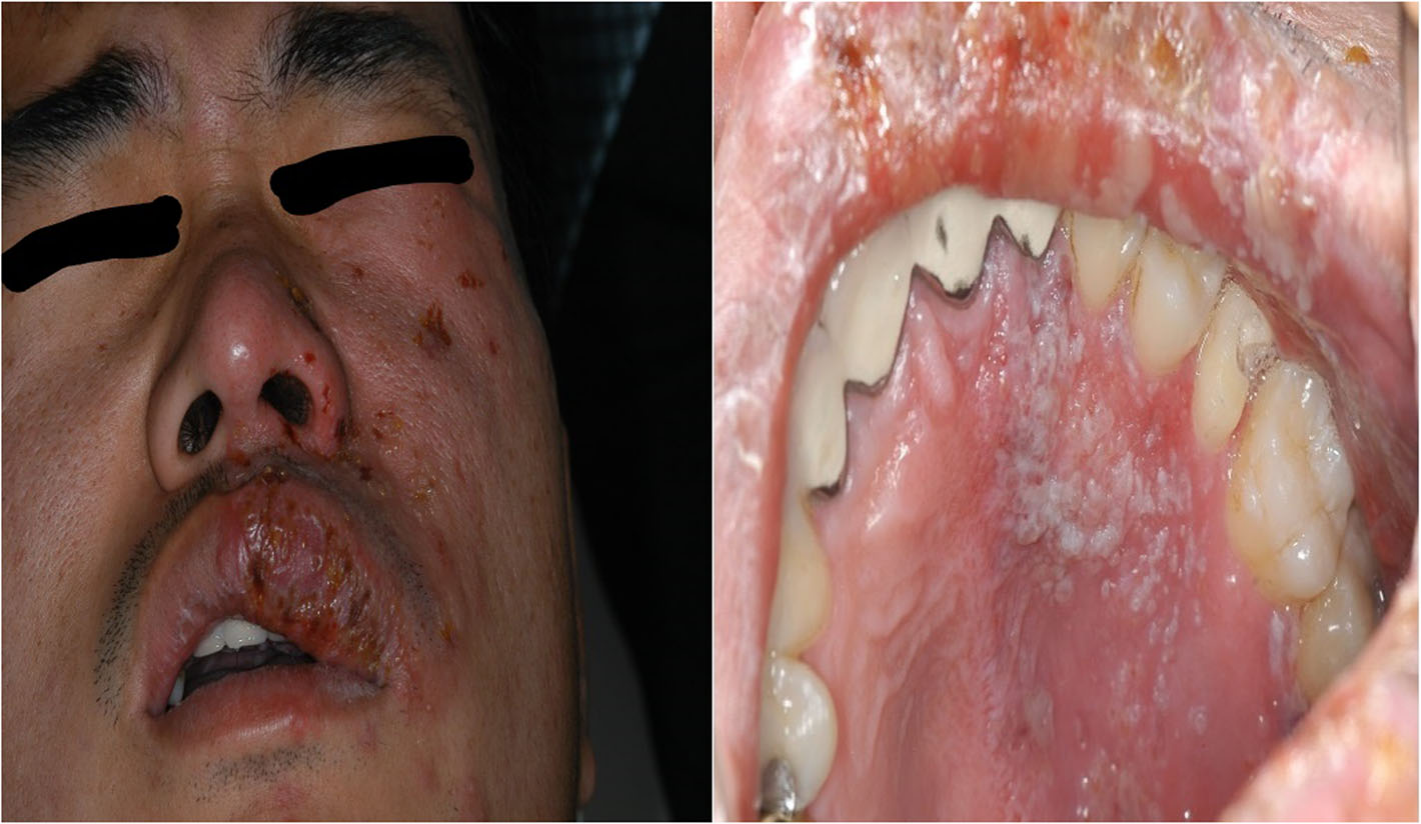
Patient complained of severe pain in the left midface area due to herpes zoster. Symptoms subsided following constant antiviral injection. (Left) Extraoral photograph. (Right) Intraoral symptoms of herpes zoster. The symptoms are restricted to the left side of the mouth

In general, symptoms of the neuralgic disorder are divided into paroxysmal and continuous; and an example for paroxysmal symptoms is trigeminal neuralgia (Figs. 9 and 10), and an example for continuous symptoms is post-herpetic pain (Fig. 11). Typical migraine, cluster headache, and temporal arteritis are examples of diseases representing vascular disorders.

### Mechanism of botulinum toxin for cranio-maxillofacial pain

Botulinum toxin is shown to be effective for related pain diseases mainly associated with a muscular disease. Particularly, botulinum toxin is known to be effective in diseases with angialgia when a muscular disease such as tension headache or muscular disease occurs in combination with the temporomandibular joint disorder. Mechanism of botulinum toxin treatment is not well known; however, some of the literature reported about the mechanism [21–26, 29].

While botulinum toxin mainly acts on the α-motor neuron, it is also known to act on the γ-motor neuron within the muscle spindles to reduce resting tone. Meanwhile, in animal testing, some researchers select a hypothesis stating that botulinum toxin has an anti-inflammatory effect, which is due to the effect of toxin suppressing the neurogenetic inflammation during headache treatment. However, more research is required to investigate the exact mechanism of toxin in relieving pain. Apart from relieving pain, botulinum toxin is also known to be useful in certain areas in the dentistry field and effective for disorders such as bruxism and clenching. This therapeutic effect of botulinum toxin is known to be due to the removal of pain caused by headache or referred pain due to hypertonicity via relieving the contraction of the masseter and temporal muscles and reducing occlusal force [21]. According to Freund’s 1999 study, the effect directly acting on the myoneural junction and the analgesic effect acting on the face and neck are all expected to play a role [30]. Some of the papers have suggested that the increase in vascularity due to muscle relaxation is more effective in eliminating trigger points or related pain than the analgesic effect [23, 25].

Similarly, if occurrence of habitual dislocation is suspected to be due to hypertonicity and disharmony in the lateral pterygoid muscle, injecting botulinum toxin into the lateral pterygoid muscle is known to alleviate habitual dislocation symptoms for a certain period of time by recovering muscular disharmony [22].

### Treatment of cranio-maxillo-facial pain using botulinum toxin

According to Binder et al., when they injected various doses to migraine patients, they found out that botulinum toxin was effective in treating migraine [26, 29]. They reported that botulinum toxin was found to be effective when they injected the toxin into the trigger points where the tender area for stimulation exists in one of the skeletal muscles, tendons, and ligaments.

**Fig. 12:**
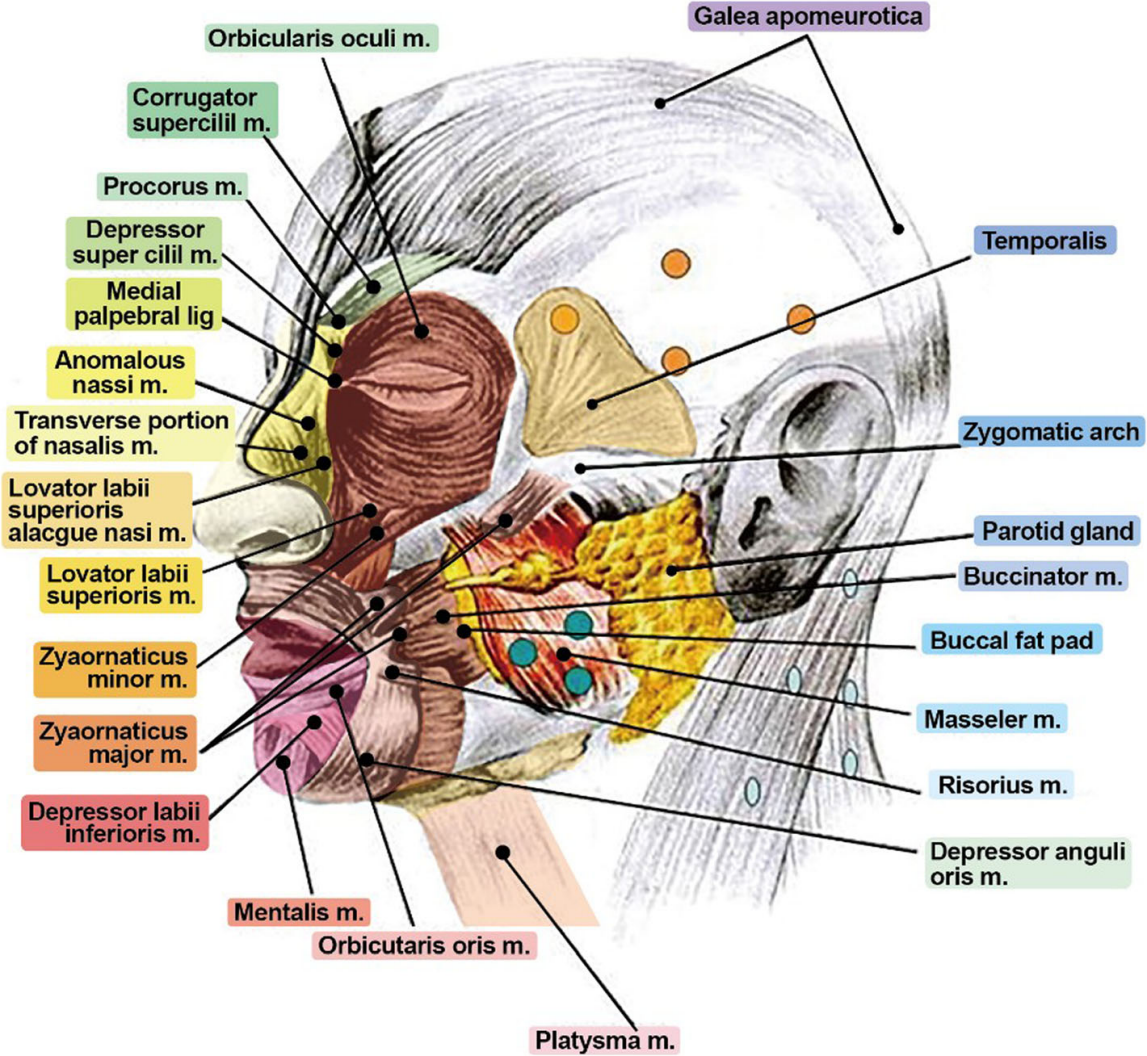
Injection points of individual muscles. The principle is to inject 5BU per injection point

Generally, areas of injection for the treatment of headache are frontal, temporal, occipital, sternocleidomastoid, and glabellar. You should inject symmetrically, and remember not to exceed 5BU (BotoxⓇ, BTXAⓇ) per injection point. It is crucial not to exceed a dose of 25BU for each major muscle. Botulinum toxin is effective when injected into the trigger points of the major muscles known to cause headache including the frontal muscle, sternocleidomastoid muscle, and four masticatory muscles. Each point is shown in Fig. 12. Table 2 represents indication of botulinum toxin injection for headaches.

**Table 2:** Candidates for botulinum toxin A therapy for headaches

1. A case that is not resolved with any other treatment	
2. Patient experiencing side effects from other treatment	
3. Contraindicated for the patient who is undergoing a standard preventive program	
4. Patient working in certain stressful environment	
5. Patient misusing or abusing drugs	
6. Patient who experienced muscle cramps in head and neck region	
7. Patient who prefers botulinum toxin injection	
8. Patient does not respond well to the standard treatment	

Contraindications for injecting into trigger zones are as follows: (1) Severe acute muscle injury, trauma, pain. (2) Patient allergic to the anesthetic agent. (3) Hemostatic disorder, bleeding tendency (diathesis), or patient taking an anticoagulant. (4) Occurrence of cellulitis in a trigger point.

Aseptic procedure and aspirating a needle are always critical while injecting botulinum toxin. Recognizing the location and shape of vessels passing through the muscles are important as well.

### Precaution of injection depending on the muscles

#### Masseter muscle

**Fig. 13:**
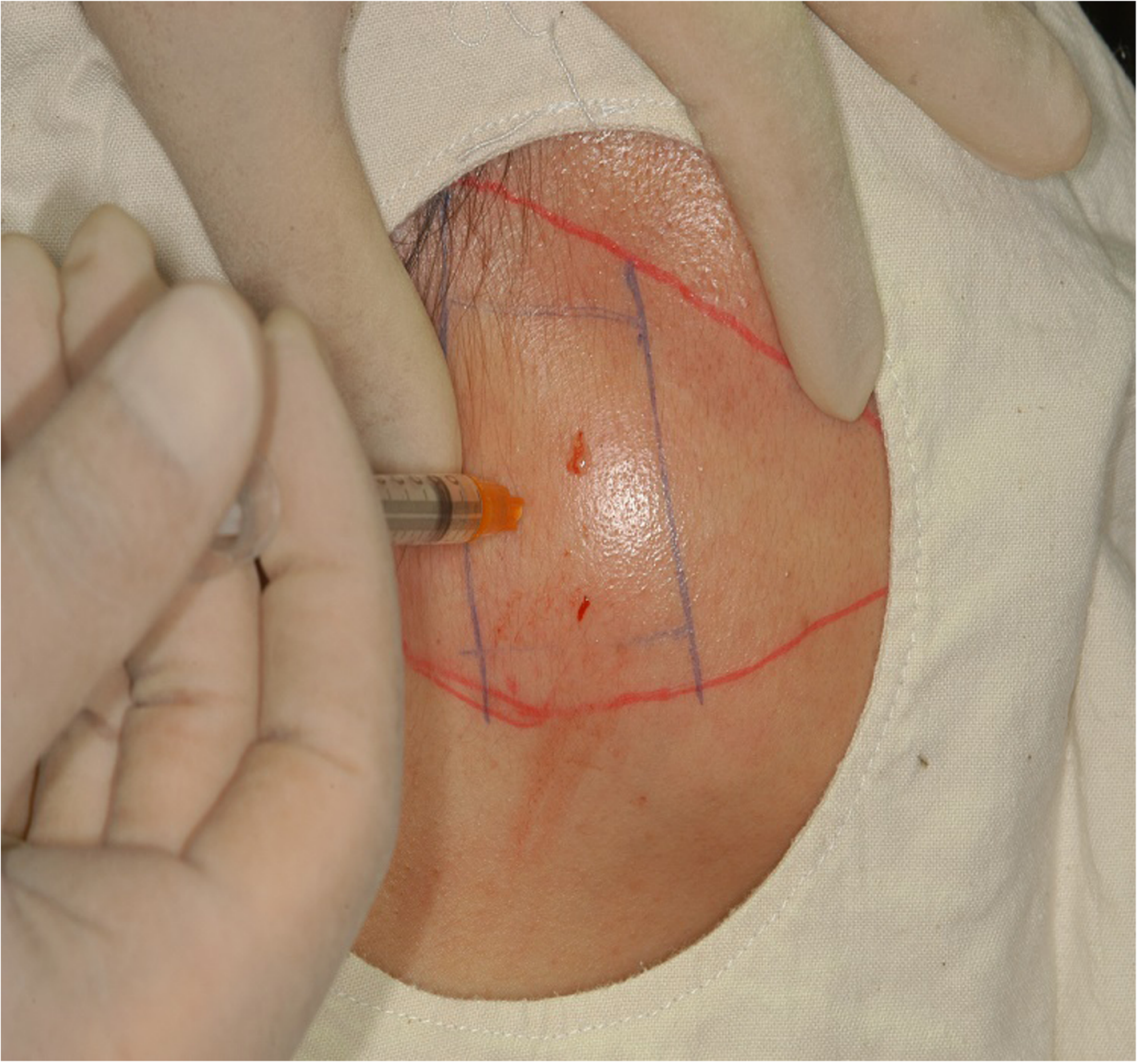
Injecting botulinum toxin A with an insulin syringe into the injecting point of the masseter muscle within the triangle

Approach extra-orally, inject into the trigger point of deep and superficial muscles with a 30G needle, watch out for facial artery. Similar to the case of square jaw patient as mentioned earlier, the injecting point is within a triangle formed by a line joining the mouth corner and tragus with the line connecting the angle of the mandible. Inject into the point 1.5 cm above the margin of the mandible and to the most bulging out portion of the muscle (Fig. 13).

#### Temporal muscle

Consider the location of the temporal artery and its branches; especially, it is advised to avoid injecting into the area near the tragus.

#### Lateral pterygoid muscle

With the patient’s mouth slightly open, while palpating the sigmoid notch under the zygomatic bone, proceed the needle toward the opposite side of the face until you find the trigger point; proceed deep into the masseter muscle. You may proceed the needle until the tip of the needle is contacted with the lateral pterygoid plate. Withdraw the needle about 3–5 mm and inject into the lower head. Be careful not to inject into the maxillary artery and buccal nerve.

### Medial pterygoid muscle

When taking the intraoral approach, enter medial to the pterygomandibular raphe, inject into the point posteriorly medial to the injection point used in local anesthesia. You can find the trigger point by moving the needle upward and downward.

#### Sternocleidomastoid muscle

Ask the patient to take a comfortable position so that the muscles do not contract, hold the muscles, and inject into the actual trigger point. Hold the entire portion of the muscles with the hand to prevent injuring the carotid sheath and adjacent structures within the anterior cervical triangle. Be careful not to induce pneumothorax by perforating the lungs.

#### Trapezius

Be careful not to induce pneumothorax by perforating the lungs. Bypassing the shoulders, inject the toxin into the trigger point.

It is recommended to inject 5BU per point for each muscle. In addition to the injection of botulinum toxin causing flaccid paralysis to such muscles, a combination of stretching therapy (passive, active elongation of muscles) will guarantee faster healing. If there is occlusion disharmony, consider using occlusal stabilization appliances and temporary administration of anti-inflammatory analgesics.

### Case report: Treatment of myofascial pain using botulinum toxin injection

**Fig. 14:**
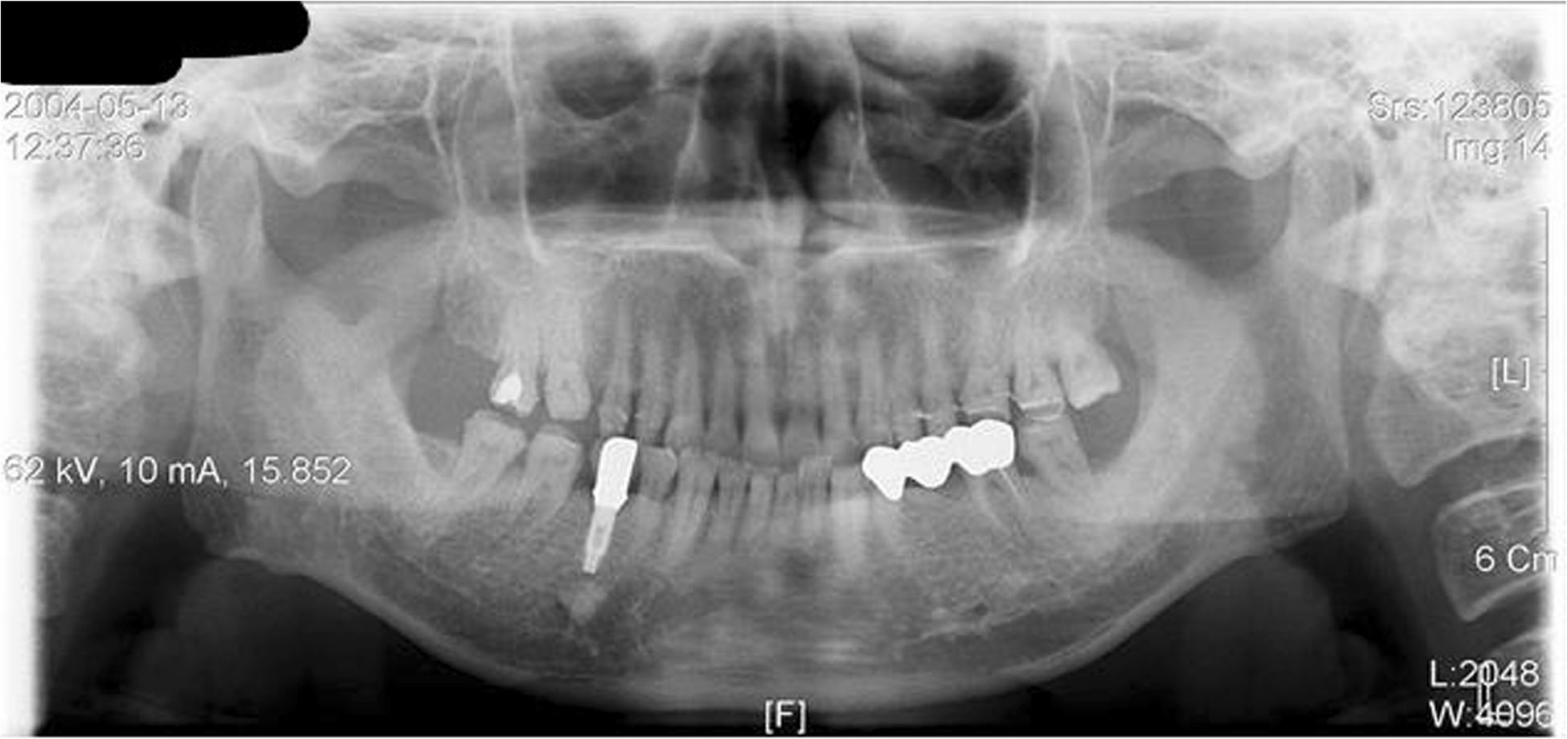
A patient who had temporary crown and bridge in the upper and lower arches complained of chronic headache and muscular fatigue due to persistent bruxism. The patient has been transferred from the prosthodontic department to our department for treatment of chronic headache, relief of bruxism symptoms, and reduction of occlusal force

**Fig. 15:**
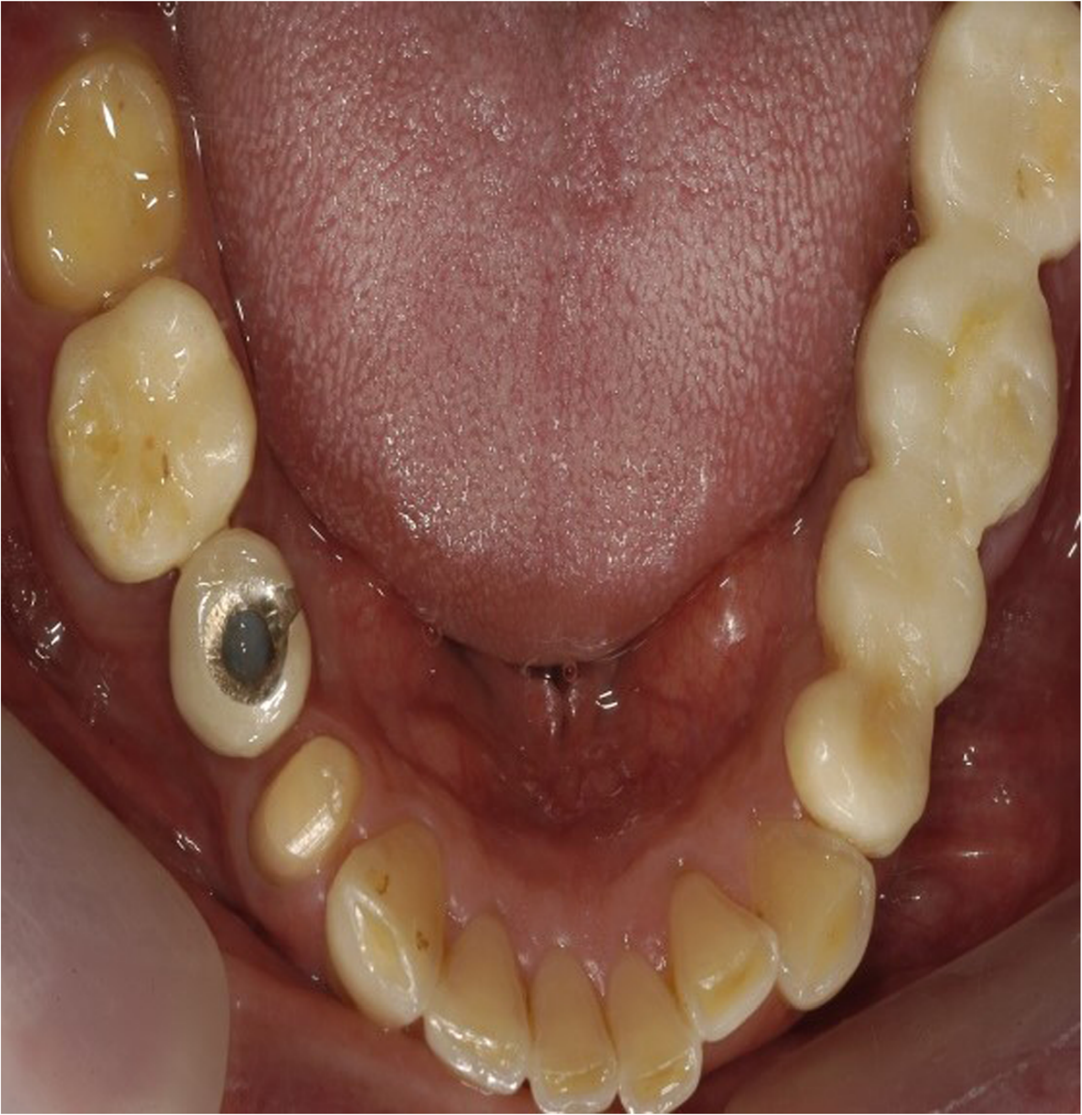
One month after the initial visit. Patient’s temporary crown remained intact and excessive occlusion was reduced

A 49-year-old man complained of headache with muscular fatigue in the morning caused by persistent bruxism. The patient was referred to our department because the patient had a temporary crown and bridge fractured that he received a week ago (Figs. 14 and 15). The patient had widespread trigger points in the sternocleidomastoid muscle and temporal muscle due to severe dystonia and persistent muscle rigidity. There was a significant improvement in the severity of bruxism and migraine after a month following injection of 20BU bilaterally into the masseter, temporal, and SCM. The patient’s temporary crown remained intact, and he did not complain about the headache anymore thereafter.

## Contraindication and side effects of botulinum toxin

Botulinum toxin treatment has less contraindication and side effects when compared with other general procedures. Ironically, problems arising from miscommunications between a patient and surgeon are the real problem. Therefore, it is always better for the surgeon to clarify with the patient beforehand if at all there is anything that the patient could miscomprehend [31–33]

For a 70-kg adult, administration of about 30 bottles of botox or BTXA at once is considered lethal. But according to the manufacturer’s instruction, administration of a maximum 5 bottles (500 U) may cause botulism. Compliance with the standard dose regimen would generally prevent the occurrence of such problems.

### Contraindication

#### Patient with mental illness

It is important to recognize a patient who has realistic expectations at the time of consultation. For the case of cosmetic purposes, consulting the patient before the procedure is of utmost important because it could eliminate factors capable of causing problems after the procedure.

#### Patient with muscular disease

The use of botulinum toxin is contraindicated in the patient with neuromuscular disease or myasthenia gravis.

#### Patient taking drugs that show drug interaction

These include aminoglycosides, penicillamine, quinine, calcium channel blocker, etc. They are known to enhance the action of botulinum toxin.

#### Pregnancy and lactation

There are no definite study results on this topic so far. But an animal study on rabbits has shown miscarriage and other side effects. Botulinum toxins are classified into Pregnancy category C drug, and there is a report that a woman who underwent botulinum toxin treatment gave normal birth to a child. There is no report of teratogenicity up to date; however, the use of botulinum toxin during pregnancy is generally not recommended.

### Side effects

So far, the most common reason for using botulinum injection is to paralyze certain muscles, and the paralyzed muscle has a limit in its function. However, for a cosmetic procedure, there are other muscles to supplement the function, or the toxin is injected to the muscle that is not functionally important.

It is most important to discontinue drug intake that increases bleeding tendency such as aspirin or NSAIDs 7–10 days before injection to prevent postoperative bruising. Inject botulinum toxin into the area where the possibility of bleeding is as low as possible and pay close attention when injecting into the area around the eyes, or edge of the forehead. While pressing on to the injection area right after the injection often leads to bruising, it helps to subside the swelling [3, 4, 6, 32, 33].

## Conclusion

Botulinum toxin is used in various ways such as temporarily resolving muscular problems in musculoskeletal temporomandibular disorders. It is used not only for treating these diseases but also for the cosmetic purpose to smoothen wrinkles caused by the muscle. The importance of knowledge on anatomical structures to reduce side effects is true.

## Data Availability

Not applicable (data sharing not applicable to this article as no datasets were generated or analyzed during the current study).

## Abbreviations

BU: botox unit: DU: dysport unit SMAS superficial musculoaponeurotic system
